# What Frailty Can Tell Us About Successful Ageing: Towards a Different Paradigm for Ageing Well

**DOI:** 10.1093/geront/gnae057

**Published:** 2024-05-25

**Authors:** Susan Pickard

**Affiliations:** Department of Sociology, Social Policy and Criminology, Centre for Ageing and the Life Course, University of Liverpool, Liverpool, Merseyside, UK

**Keywords:** Existential phenomenology flourishing, Holistic approach

## Abstract

This paper considers the concept of successful aging by means of a dialogue with the concept of frailty. This dialogue reveals the limits and blind spots of both concepts as well as their apparent dichotomy and the importance of understanding both the objective and subjective experience of aging. In particular, the dialogue highlights that both frailty and successful aging are social constructs that derive their meaning from what society values (autonomy, youthful capacities). They cannot by themselves account for the fact that flourishing and frailty are often found together whilst conversely successful aging may not bring satisfaction. I argue that the arts and humanities supply us with conceptual and methodological tools with which to revisit what it is to age well offering a holistic approach that combines sensitivity both to older people’s lived experience and to the underpinning material conditions and embodied realities. For this purpose, and building on previous scholarship in cultural gerontology, I suggest the unique value of concepts and frameworks associated by Simone de Beauvoir. When brought into alignment with the tenets of cultural and narrative gerontology, these concepts facilitate a rich understanding of the nuances and paradoxes of flourishing in deep old age which can also aid in restorying both old age and the life course more generally. I illustrate this through two examples: a feminist self-help guide to aging, which accepts both limitations and growth and a film that imagines the possibilities of authentic aging even despite a context of poverty, patriarchy, and Alzheimer’s disease.

## Background: The Secret to Successful Aging?

In her third volume of memoir, the brilliant graphic artist Alison Bechdel, who has explored very personal themes in her autobiographical work, turns to what we might see as a meditation on successful aging. In it, Bechdel reflects on her life-long obsession with exercise, from a personal and philosophical perspective. The desire she has for mastery of her body is at once a desire to overcome death and to assert herself as an individual separate from her parents. As a preteen, at the same time as she starts to fear her parents’ dying, she orders her first martial arts guide entitled “The Secret to Superhuman Strength.” As she enters her teens, then her 20s and 30s, she takes up running, karate, mountain biking, cross-country skiing, each test more strenuous than the last, and with each goal accomplished she asks herself: “Had I found it? The secret to superhuman strength?!” The goal is a version of the self which she describes thus: “Oh, to be self-sufficient! Hard as a rock! An island!” (2021, p. 8). The aspiration for superhuman strength is the ideal of progress to her; cycling uphill is progress literally and downhill decline and she observes: “If I had to choose between only riding downhill or only riding uphill for the rest of my life—an existential question that I pondered often—I would take the uphill.” Why? The answer is this: “I was in control. Careering downhill, who knew what the next moment would bring.” And yet with each athletic achievement (she runs super-marathons) and professional triumph (her book is a New York Times bestseller and she wins a MacArthur “genius” Award) she still uncertainly asks herself: “Had I discovered it at last? The secret to superhuman strength?” Whatever it is, it eludes her. As she grows older she associates strength (psychological and metaphysical) with physical strength and worries about getting weaker, that her “illusion that I might somehow stave off death” is unraveling, p. 9). Her response is to try harder. Yet still her search for superhuman strength is never realized; moreover, all her success brings her no satisfaction; one goal is simply replaced by another in an endless linear pathway focused on outcomes.

This struggle for success via mastery of the self reflects a key value of late modernity, building on a theme of self-discipline traceable back to Plato, who sees the good life as mastery of the self, meaning the self can distance itself from and objectify both the external world and one’s response to it (including one’s body). Plato’s vision of reason in an ordered cosmos has, in modernity, become one of instrumental reason, efficacy, consistency, and objectivity. In contemporary times, “success” as a concept is particularly associated with a seamless unity between economy and society. To be entrepreneurial is to be successful and “success” defines “the good life.” In terms of neoliberalism, the emphasis is on technologies of active self-care, in which each individual became an “entrepreneur of him -or herself” (Rose, 1999, p. 164) that requires one to be “in continuous training, life-long learning, perpetual assessment, continual incitement to buy, to improve oneself, constant monitoring of health and never-ending risk management” (1999, p. 254). This ethos is evident in the concept of “successful aging” as it has been popularized in the work of Rowe and Kahn (1987, 1997, 1998). Successful aging aligns itself with “progress” measurable by the presence of excellent health, with no underlying disease or functional limitations, social engagement and productivity, and the absence of disease or risk of disease. Implicit in this discourse, moreover, is an imperative to control and to flexing the will very much in the mode of Bechdel’s search for superhuman strength. Noting that they regard aging “as largely under the control of the individual” (1998, p. 37), Rowe and Kahn’s aim in their best-selling self-help guide is to “pinpoint the many factors that conspire to put one octogenarian on cross-country skis and another in a wheelchair” (xii) and consequently offer their advice in a series of chapters advising on the steps one can avoid to maximize good health and avoid disease and disability. Success for them is “flourishing” (p. 37) yet, as the underlying message of Bechdel’s graphic memoir makes clear, this can feel hollow; focused on achievement, the sense of accomplishment, as with all goal-related activity, is fleeting and quickly evaporates, priming one to reach for the next goal, and thereby blocking out the fuller meaning of the journey through the life course. Indeed, through a series of breakups, parental deaths, exquisite glimpses of a cosmic consciousness through losing herself outdoors in the wilds, Bechdel is haunted by the sense of an alternative path running parallel to this one, that emphasizes different truths and values. Sarah Lamb captures this sentiment for the latter part of the life course in her question: “Does the currently prevailing successful aging model overemphasize independence, prolonging life and declining to decline at the expense of coming to meaningful terms with late-life changes; situations of (inter)dependence, possibilities of frailty, and the condition of human transience?” (2014, p. 42). The answer is surely “yes.”

Thus, in the next section, I center the condition of vulnerability at the heart of aging, via a comparison of the concepts and categories of “successful aging” and “frailty” respectively in order to further beam light into the blind spots of the successful aging narrative and what it leaves untold.

## What Can “Frailty” Tell Us About “Successful” Aging?

Over the decades, many models for operationalizing successful aging have emerged (e.g., Bowling & Dieppe (2005); also see Annele et al (2019) for a current review). These models have developed in parallel to measures of frailty, of which the latter incorporate increasingly sophisticated scales and indices (e.g., Rockwood & Mitnitski, 2007) and which respectively correspond to the two dominant narratives of progress and decline (Gullette, 2004). However, one’s subjective feeling of aging well, a sense of wellbeing and good health, which extends beyond these objective measurements, is not exclusive to those who are “successfully aging” in Rowe and Kahn’s terms (e.g., Strawbridge et al, 2002). Looking at frailty, it has been known since the work of Mossey and Shapiro (1982) that a positive sense of subjective wellbeing, regardless of measurable factors, is a “more reliable predictor of adverse outcomes than that afforded by only considering medical and psychiatric diagnoses” (Powell, 1997, p. 24). In this sense, frailty and flourishing often coexist, a fact that challenges the tenets of successful aging as well as its claim to be the primary definition of aging well. Bowling and Dieppe point out, ‘there is ample evidence that many elderly people regard themselves as happy and well, even in the presence of disease or disability’ (Bowling & Dieppe, 2005, p. 1550). Similarly, ethnographic research has suggested that there is a disjunction between being assessed as frail and *feeling* frail, with a range of social and existential factors underpinning the latter, such as the loss of the ability to pursue tasks of significance to one’s identity or the loss of key loved ones (Grenier, 2006; Kaufman, 1994; Nicholson et al 2012; Warmoth et al, 2016). Earlier conceptualizations of frailty highlight the “balance” (between assets and deficits) that is key to understanding both good health and frailty in later life (Rockwood et al, 1994) rather than positing a clear division between them. Similarly, whilst Bowling and Dieppe and others list a number of features present in aging well, making lists of assets and deficits, positive and negative resources, these measurables cannot by themselves capture the balance that is at the heart of this experience. For example, in Ebrahimi et al’s study, older people who were clinically frail could nevertheless feel their health to be excellent if a certain sense of balance or coherence (Antonovsky, 1987), which was uniquely situated in biographical terms, was attained. This included being able to do small things that matter to them, whether it be moving from the toilet bowl to the wheelchair in the case of an older person or preparing oneself breakfast. Ebrahimi et al observe: “The threshold for the experience of health and being in harmony and balance not only varied for the same person over time, but it was also different for different people” (2012, p. 1517). This balance has been described by Baltes and colleagues (1990) in terms of psychological self-development with those more successfully aging able to employ “selective optimization with compensation” and by Tornstam (1997) as requiring an opening oneself up to different values and meanings than those that apply in earlier life.

A more fundamental point is that both successful aging and frailty are social constructs (Richardson et al, 2011); in other words, not natural categories, but shaped by cultural values, such as the contemporary emphasis on autonomy and productivity (see Pickard, 2014), as well as assessments of aging bodies made through the prism of the decline narrative (Katz, 1996). As ways of conceptualizing complex situations through the framework of particular epistemologies (e.g., medicine and social science respectively) these constructs run the risk of reifying these lived situations, congealing them into “things” and thereby enabling a distancing process to take place, a distancing that lies at the heart of the Enlightenment model of science which aims to objectify and measure the world. Whilst not in itself problematic, and in many situations, indeed, remarkably helpful and important, this approach does have blind spots. Importantly, however, these blind spots are not restricted to science alone. For example, Chris Gilleard (2023) observes that dominant approaches within the sociology of aging mostly bracket out both biological and lived experience, focusing instead on structures, institutions, and discourses: what he calls the “externality of aging and old age” (2023, p. 9).

Certainly, within the social sciences’ academy today poststructuralism/postmodernism is enjoying particular popularity, with Foucauldian approaches perhaps most significant among these and with the consequence that the fleshy, corporeal reality of the aging body ceases to exist in its own right but is viewed rather as the product of such discourses. By contrast to the sociology of aging, however, the unique contribution of cultural gerontology lies in its focus on meaning and subjective experience and agency over structure. In addition, it foregrounds theory, embodiment and diversity/heterogeneity (although it has been applied less to deep old age; Twigg & Martin, 2015). Teasing out some of these dimensions, Twigg (2004, p. 70) stresses the importance of both the biological and agentic (contra Foucault) dimensions of the lived body and highlights the importance of a dialogical relationship between theory and experience, in order thereby to avoid the perils of biological reductiveness and social determinism as well as simple prima facie acceptance of the “truth” of experience. The question of how exactly to conduct and maintain this dialogue lies, of course, at the very heart of the matter.

## How to Understand “Aging Well”: The Contribution of the Arts and Humanities

In this section, I suggest that a rich potential source for such a dialogical approach lies in the theories and literature of the arts and humanities which are well placed to illuminate the holistic experience of aging well. To do so, I suggest that a fruitful approach is provided by the existentialist phenomenological method, building on previous work in this area. For example, Laceulle (2018) has emphasized the existential precept of authenticity, drawing on Rousseau, Heidegger, and Kierkegaard among others, as has Dohmen (2013) and Baars (2017), using this approach, moreover, to highlight the importance of vulnerability and finitude and to critique the concept of “successful aging.” The particular contribution of this paper lies in my suggestion to employ concepts and frameworks developed by Simone de Beauvoir within this existentialist tradition, which are somewhat lesser known in cultural gerontology, although highly complementary to its aims, methods, and conceptual approach. Through these unique concepts, it enables access simultaneously to lived experience as well as the structures that frame such experience and can combine multiple dimensions whilst at the same time offering a richly layered perspective on choice, flourishing, and individuality which contrasts sharply with that of the discourse of successful aging.

The first such concept I highlight here is that of the embodied “situation.” The situation is related to but distinct from, the “standpoint” of feminist theory, and it is more philosophically developed than the term “context” alone implies. It also mediates between freedom and “facticity,” which are the terms Beauvoir uses to distinguish between the elements of our lives that we choose and those elements that are ascribed or imposed (such as our body, age, gender, class, sex, and so on) respectively (Tidd, 2004, p. 30). As such it can account for “an embodied subject […] that, although never an absolute freedom or pure consciousness, has a viewpoint on the world and an intentional relationship with it” (Kruks, 2001, p. 61). A focus on the “situation” facilitates insights not just into individuals but into the groups and collectivities to which individuals belong and whose experience is structured in similar ways. Indeed, in Beauvoir’s very sociological philosophical vision, individual situations cannot change without more substantive change occurring at the collective level (Beauvoir, 1997). Beauvoir’s phenomenological existentialism mediates between the concrete and the abstract, the particular and the general and thus allows a multilayered insight into experience as it is framed within, but not wholly created by, social structures.

Indeed, central to this philosophy is the emphasis on agency (as equally, if not more, important than structure) as expressed through choices and a key concept for making sense of this is “authenticity.” For Beauvoir (1976), authenticity is a form of active self-making involving “transcendent” projects, that is, those plans and intentional activities that surpass the given situation. Authenticity involves choices made freely in the pursuit of projects of value to the individual that gives life meaning, rather than a succumbing to external pressure or ideologies (entailed in regimes of power). The reverse of this is not determinism but ‘bad faith’ whereby individuals make choices while denying (to themselves, chiefly) that they have any such choice, such as adopting a highly gendered mode of life when other options are open, acquiescing in the values of neoliberalism, and so on. In her text, *The Coming of Age* (1970) Beauvoir describes certain individuals who continue to fulfill projects deep into old age. Her sociological point in this text is that these individuals can do this because they enjoy privileges and advantages unknown to ordinary men and women and indeed she was somewhat bleak about the possibility of transcendence for the working classes (doubtless accurate at the time in which she was writing). However, where the situation is less damning for many working-class older people today, poverty, frailty and inequality still remain which, together with bodily impairments, constrain the possibilities of transcendence in old age. Yet this does not deny authentic aging as Kruks (2024) asserts, helpfully drawing out a muted theme in Beauvoir, which concerns the authenticity that may be associated with less transcendent projects. Indeed, she suggests that we can, and indeed should, reconceptualize the concept of the “project” in order to incorporate a broader range, including those belonging to the everyday, and even tiny but meaningful acts undertaken in the context of powerful constraints. These include the actions one needs to thrive in very difficult circumstances, such as moving between the toilet bowl and wheelchair and so on in the example noted earlier (Ebrahimi et al, 2012).

A third key concept deriving from this philosophy is that of ambiguity, which arises from the human condition as being both embodied and fleshy and yet experienced through the lens of society and culture. For Beauvoir, human life is comprised of tensions that are not resolvable—for example, between having transcendent projects and yet being mortal, being a subject for oneself and an object for others—and in terms of aging being both a body measurable through the tools and tests of biomedicine and an embodied being for whom certain things matter deeply. Every aspect of life is riven with ambiguity, which Beauvoir urges us to accept, in all its messy uncertainty, in contradiction to the dominant philosophical imperative of modernity to classify in one category or another. This insight has been applied to frailty by Gadow (1983) noting the ambiguity and contradictions in frailty that meant that frailty is at once strength and the opportunity to turn the object body into the subject body (see also Pickard, 2024).

Applying the concept of the “situation,” structured by an ontological ambiguity and a tension between choice and constraint, to aging and old age enables us to integrate elements connected to the “externality” of age, including social structure and physiological condition, as well as to “inner” experience as grasped through stories of aging well. Furthermore, it brings a clarity and focus to the concept of “lived experience” which is often used loosely and can sometimes detach itself from structural and historical locations (McIntosh & Wright, 2019). This approach also resonates powerfully with the ethos of narrative gerontology. Using terms derived from existentialism, Kenyon and Randall (1999) describe how the “epistemological assumption of narrative gerontology… is that life stories are made up of both facticity and possibility.” They explain: ‘Facticity refers to the story we *are* at any one point in time ‘which includes gender, race, location, and general other biographical factors. “Possibility, on the other hand, refers to those elements of a life story that are subject to change, or to *restorying”* (1999, p. 2; original emphasis). Sharing Beauvoir’s emphasis on freedom, what is particularly liberating about narrative gerontology is that “we can never know in advance what belongs to a person’s facticity, and is therefore locked in, and what is capable of being restoried through their sense of possibility. In this respect, narrative gerontology enables us to explore the complex dynamic between our inner personal meaning and the constraints that are imposed on us from outside” (Randall & Kenyon, 1999, p. 2); and, I would add, by the aging body itself.

This perspective helps us to grasp the complexity of aging well including flourishing in conditions of constraint. In the next section, I illustrate this through two brief examples focusing on the subjective experience of aging well. My first example is a feminist guide for aging by the psychologist Mary Pipher, *Women Rowing North* (2019) which views later life as a new and valuable life stage as well as an opportunity to develop (including out of constraining gendered roles). My second is the movie *Poetry* in which, through the radical imaginative reach of the writer and director, Lee Chang-dong, an old woman is depicted as flourishing whilst simultaneously occupying a disadvantageous social and economic situation and being in the early stages of Alzheimer’s disease (AD).

### Women Rowing North: Navigating Life’s Currents and Flourishing as We Age by Mary Pipher

Mary Pipher, the self-proclaimed feminist writer of the best-selling psychological text about the tensions of youthful femininity, *Reviving Ophelia,* turns her attention to aging in this self-help guide. She describes her aim in this book to be similar to that in *Reviving Ophelia* in that it “explores a specific life stage from a feminist perspective, revealing the reality of women’s lives as opposed to the dominant cultural stories about us” (p. 4). As such, the guide is a map for an ongoing journey rather than a destination. Pipher is sanguine about this journey declaring: “Women in their sixties and early seventies are crossing a border and everything interesting happens at borders” (p. 3). The message is that once the situation of later life is embraced, rather than denied or resisted, it opens up unimagined possibilities to accept it as a stage of some value and meaning.

The text advises women to shift researcher response to aging away from the pernicious decline narrative and assume the possibility of qualitative growth through the continuation of projects. But this is far from offering a straightforward progress narrative. Pipher writes: “Attitude is not everything, but it is almost everything. In fact, in many situations, it is all we have. Especially as we age, we can see clearly that we do not always have control, but we do have choices. That is our power” (2019, p. 2). Pipher treats older age as a stage of life replete with all the ambiguities, challenges, and opportunities of other life stages. Placing this journey in a life course perspective she points out that all life stages bring challenges and also rewards. Some life stages (not necessarily the later ones) will offer more challenges than others. But again, “attitude and intentionality are the governors of the process” (p. 19). She suggests accepting both joy and suffering as underpinning our growth: using the language of the philosophy of ambiguity, she declares that this approach embraces paradox: “we can describe ourselves as living in ‘both/and’ terms” (p. 20). Both loss and gain are intimately connected in this life stage and comprise a unique balance, or what Pipher calls an “amazing calculus” (p. 23). She adds: “We learn to accept what will become a constant cycle of maintenance, loss, accommodation, and renewal” (p. 42). She includes the challenges of the aging body firmly in her account but emphasizes the necessity of achieving balance on our own terms: ’We suffer a setback, we regain homeostasis, and soon we are enjoying our lives again’ (p. 52).

Our cultural response at all phases of life is to classify and categorize rather than indulge ambiguity. However, in lived experience: “emotions frequently occur in combination such as sorrow and rage, anger and fear, or love, sadness, and bitterness, all at the same time” (p. 56). For older women, for whom this guide is written, strength may also involve challenging traditional gendered roles that have left us emphasizing being “nurturing and available to others” (p. 100), rather than developing self and meeting our own needs. Strength requires authentic choices, if necessary stepping out of that mindset and those roles if and where they leave us without space for our own requirements. Like Beauvoir, she places a premium on freedom and authenticity. She defines freedom as “the ability to make conscious choices in accord with our deepest values” (p. 115), rather than reacting to events (and responding to others’ demands). However, as in Kruks’ reworking of Beauvoir, such projects can be limited but still meaningful and satisfying. For example, she explains how one woman’s identity shifted from a life-long focus on professional success to capacity to care both for her beloved husband of many years as he developed Parkinson’s disease and for herself during the challenging times they faced. Pipher foregrounds her own standpoint, moreover, including auto-ethnographic examples too: for example, she recounts how she slowly relinquished her life-long identity as a rescuer and carer of others: “Could I lose this no longer functional habit and simply be present?” (p. 157), she asks herself, reflecting on a day spent enjoying the beauty of doing very little, for once, except relaxing in beautiful surroundings and being present to all this brings in a gerotranscendent sense. There is a significant contrast here with the emphasis on activity and productivity found in successful aging. Again, most of all, aging is characterized by a number of antinomies that Pipher does not suggest should be resolved, such as the way that vulnerability and frailty can foster flourishing: “Without suffering, too much is taken for granted. With a transcendent response to suffering, nothing is too small to appreciate. We can enjoy every fresh apricot, blazing October day, and visit with a friend. We can be awake and whole” (p. 23).

### Poetry (2010) Directed by Lee Chang-dong

In this remarkable South Korean film, a story is told that suggests how, in conditions of extreme structural disadvantage and ill-health, as our older protagonist, Mi-ja is diagnosed with AD, she learns how to express (and assert) herself, exhibit authenticity and indeed flourish for what seems likely to be the first time in her life. We can trace this narrative arc through two key themes. The first is Mi-ja’s aim of writing a poem for the first time in her life, thereby fulfilling a girlhood potential that, amidst the many onerous roles of adult life, she never found the space to realize. The teacher of the evening class she attends tells the poetry students that in order to write they must learn to “really see” the world around them. As she meticulously does her homework, carrying around a notebook to record her observations, Mi-ja gradually learns to see the world and in turn this brings clarity to all aspects of her own situation. She moves from seeing the apple she holds in her hand, the ripe apricot crushed on the path where it has fallen from the bough, the blood-red flower growing outside the window, slowly outwards to a clear perception of her own place in the world, involving recognition of what she has endured, suffered and enjoyed and who and what she values now. Her new understanding—which proceeds through flashes of poetic insight, rather than rational analysis—fills the present with the utmost significance and value, especially as her memory fails her and the past fades away. By the end of the film she succeeds—as do none of her fellow writing classmates—and despite her increasing AD, in composing her poem. This is an authentic goal, freely chosen and fulfilled; it suggests that the diagnosis of AD does not define the totality of her experience of old age which remains first and foremost an existential situation not reducible to a property of the brain.

The second theme of the movie depicts Mi-ja’s dawning realization of the oppression she has endured and in turn her fighting back. This insight begins when she learns that her rude and lazy grandson, who lives with her whilst his mother gets on with her own life unencumbered in a different city, was involved in a gang-rape of a schoolmate, Agnes, which led to the girl’s suicide. Although sympathizing—and indeed deeply empathizing—with Agnes, at first she does her best to collect her portion of the “bribe” (on the instruction of the fathers of the other boys, who are doing the same) in order to attempt to dissuade Agnes’ family not to report the boys to the police. She obtains her share by having sex with the older disabled man for whom she works as a carer, asking him afterwards for the money in a way the audience senses is completely out of this modest and dignified woman’s usual character. Agnes’ grieving family reject this bribe, however, and so the fathers send Mi-ja to meet Agnes’ mother in a last-ditch attempt to persuade her to accept it. However, during an encounter with the mother in the fields where she is working, Agnes does not do this. Instead, entranced by the vision of crushed apricots that have fallen in their ripeness from the trees onto the ground, (and in a scene reminiscent of the section in Pipher), she leaves her sense of duty aside and instead speaks to Agnes’ mother only of this beauty. After this, when she returns home she reports her grandson to the police and obliges her daughter to take responsibility for her own son—attaining, amidst the undoubted suffering of her situation, a vibrant freedom and transcendence. The movie suggests to the audience that we, like Mi-ja, need to learn to “really see” and in so doing to really see old age, in all its intricate mosaic, not as a younger person views it but from the inside. To do so, the movie highlights the role of ambiguity working in a subtle and nuanced way to turn certainties into more equivocal states akin to Tangazaki’s insights on Japanese aesthetics. Thus, for us as observers, the inner experience of Mi-ja’s aging is reached indirectly by the way one thing is reflected through and by another, “not in the thing itself but in the patterns of shadow, the light and the darkness, that one thing against another creates” (1977: 30, quoted in; Danely, 2023). Hence, a focus on vulnerability brings the outline of strength into view and vice versa. We can see here how, as Pipher advises and the tenets of narrative gerontology prescribe, freedom can lie in restorying elements of one’s life even when facts remain unchanged.

The movie offers a dementia counter-narrative (Chivers, 2013), however, it provides at one and the same time also a counter-narrative to that of successful aging. Firstly, it is through Mi-ja’s faltering health and existential crises, both of which she faces courageously, that she finally finds the means to fulfillment and self-realization. Secondly, “failure” in a more powerful sense is clearly revealed to lie in the behavior of others and in the structures of an ageist, sexist, and consumerist society, making her story hugely political as well as quietly powerful.

## Discussion and Concluding Remarks

This paper has suggested that, as with the mythical search for superhuman strength with which I began this account, there is no secret to successful aging to be discovered and imparted. Rather, we have no choice but to accept and live our lives in the understanding that, as human beings, we have projects and may enjoy good health and favorable conditions but we are also mortal which makes us vulnerable and finite. If we have not grasped this existential truth before, old age is a time to reflect on it and to savor the insights and clarity of vision it permits. The particular framework I have suggested here draws from Beauvoir’s existential philosophy together with narrative gerontology which is adroit at capturing holistically the experience of aging and old age, in its good and bad aspects. The shift in emphasis away from “success” also offers alternative ways of attributing meaning to the rest of the life course. For example, whilst Tornstam’s concept of gerotranscendence is important and valuable there is no reason it should be restricted to the latter portion of the life course. Returning to the example with which I began this paper, Bechdel discovered this for herself when, after a succession of relationships ended as, inevitably, her girlfriends decide that they cannot keep up with her frenetic pace in pursuit of superhuman strength, she takes a vacation with her new partner, enjoying walks and beach holidays for the first time ever. This leads to a moment of epiphany. Instead of striving to complete goals she realizes that the moment of importance is here and now. She slows down and savors the moment of being alive, imperfect as it is, with her aching limbs, bad back, and unrealized ambitions. Although she knows she will continue to push herself in all ways, she accepts that “nirvana *is* samsara” (2021, p. 232) and that the sources of living and aging well are to be found in the everyday, the savoring of apricots, so to speak, as well as the accomplishment of goals, a realization that is as simple as it is profound. As “successful aging” is part of a broader discourse of success that claims every other life stage in different ways, a new imaginative appreciation of old age (including deep old age) offers important possibilities for reconceptualizing, healing (in the etymological sense of making whole) and re-enchanting the life course itself.

## Data Availability

As this paper uses philosophical arguments, drawing on secondary texts only, the TOP Guidelines regarding replicability of data do not directly apply.
